# Health State Utilities for Patients with Brain Metastases

**DOI:** 10.7759/cureus.667

**Published:** 2016-07-04

**Authors:** Nataniel H Lester-Coll, Arie P Dosoretz, James A Hayman, James B Yu

**Affiliations:** 1 Radiation Oncology, Yale University School of Medicine; 2 Radiation Oncology, 21st Century Oncology; 3 Radiation Oncology, University of Michigan Health System

**Keywords:** utilities, quality of life, Stereotactic Radiosurgery, whole brain radiotherapy, brain metastases

## Abstract

Purpose: Estimating the cost-effectiveness of whole-brain radiation therapy (WBRT) and stereotactic radiosurgery (SRS), including Gamma Knife radiosurgery (GKRS), requires the quantitative measurement of patients’ health states after treatment. We sought to quantify individuals’ preferences for the relevant health states after WBRT or GKRS for brain metastases on a 0 to 1 scale, where 1 is perfect health and 0 is death.

Methods: We prospectively measured utilities in patients with brain metastases evaluated at Yale for consideration of WBRT and/or GKRS, as well as oncology nurses who had cared for patients with brain metastases before and after WBRT or GKRS, using the Standard Gamble (SG) technique. Demographic information was also collected. Nonparametric tests were used to compare potential differences in utility values and for subgroups based on demographic characteristics.

Results: There were 24 patients and 31 nurses who completed the study between December 2013 and May 2015. Median utilities ranged from 0.85 for the status-post (S/P) GKRS state to 0.25 (for neurologic dying). The median utility of being S/P WBRT was 0.70 compared to 0.85 S/P GKRS (p < 0.001). The cognitive decline from WBRT was associated with a notably low utility score of 0.30. There were no statistically significant differences between patients’ and nurses’ median utility scores.

Conclusions: These SG utilities provide unique insights into brain metastases-related health states from the patient and provider perspective. As perceived by individuals with direct knowledge of the health states in question, WBRT has a significantly lower utility compared to GKRS. Cognitive decline following WBRT is associated with significant perceived reduction in quality of life. Differences in the relative importance of overall survival and quality of life with treatment existed between patients with different stages of disease. These utilities can be used to calculate quality-adjusted life expectancy in cost-effectiveness evaluations of SRS and WBRT.

## Introduction

The incidence of brain metastases, the most common intracranial tumor, is rising [1-2]. Standard local management options for brain metastases consists of surgical resection, whole brain radiation therapy (WBRT), and/or stereotactic radiosurgery (SRS), including Gamma Knife radiosurgery (GKRS). Randomized controlled trials comparing these modalities have demonstrated similar survival and improved neurocognitive outcomes associated with initial SRS alone in appropriately selected individuals with up to three to four metastases [3-6]. Due to the improved neurocognitive outcomes associated with initial SRS, many centers advocate for SRS as the initial therapy for brain metastases. However, given the potential expense of SRS and lack of associated survival benefit, it is unclear whether the potential cost of SRS is justified based on neurocognitive benefit alone. Therefore, cost-effectiveness studies comparing WBRT and SRS are sorely needed.

Cost-effectiveness analysis offers an analytic method to evaluate treatment outcomes, patient preferences, and resource utilization. It relates costs to outcomes by calculating the ratio of cost per unit of effectiveness, such as the cost per year of life gained [7]. Although a difference in survival is an intuitive measure of effectiveness, it fails to capture the potential change in the quality of life associated with cancer treatments. The impact on quality of life associated with cancer therapy is particularly important when survival gains are modest. Similarly, relying on survival outcomes alone is not useful when a given treatment option has no clear survival advantage. Therefore, in an attempt to simultaneously account for quality of life and survival, cost-effectiveness studies combine survival and quality of life into a single quantity called the quality-adjusted life year (QALY) [8]. A QALY represents a year of life gained that is weighted by the patient’s quality of life during that year, typically quantified as a “utility.” A utility is a measure of a patient’s preference for a given health state, on a 0 - 1 scale where 1 is perfect health and 0 is death [9]. Measurement of utilities is, therefore, critical in evaluating new health technologies as health care costs continue to escalate [10].

The majority of published analyses comparing outcomes from WBRT and SRS do not factor in patient preferences [11-18]. Those studies that do incorporate utilities use values based on expert opinion only [16-19]. However, there are currently no direct utility measurements in patients with brain metastases. By directly measuring patient utilities for a range of possible outcomes following WBRT or SRS, it is possible to quantify the trade-off between recurrence risk and side effects and ultimately help decide whether WBRT or SRS is preferred. Therefore, the current study was undertaken to directly develop and quantify utility values for health states that can be experienced after WBRT or SRS for brain metastases.

## Materials and methods

This study was granted exempt from review by the Yale University School of Medicine Human Investigation Committee (HIC Protocol # 1308012625) under category 7: Research Involving Response to Non-Physically Invasive Stimuli. Written consent was not required for the study. However, oral consent was obtained for every participant, including an overview of the survey and the kind of information that would be recorded. We surveyed two cohorts of subjects: patients with brain metastases seen in consultation for WBRT and/or GKRS at the Yale Department of Therapeutic Radiology and nurses in the Yale Departments of Therapeutic Radiology and Medical Oncology. Nurses were selected if they cared for patients with brain metastases before and after WBRT or GKRS. Since our department uses GKRS and not other forms of SRS, we used descriptions of GKRS specifically in our survey. Cost-effectiveness analyses are used to make societal decisions; therefore, one might imagine that surveying random members of the public would be the ideal cohort to study. However, in practice, it would be difficult to educate such people about the health states and, therefore, healthcare providers are often used instead. In our study, patients and nurses were enrolled because these populations have direct knowledge of the health states in question but also are known to perceive the same health states differently [20]. In keeping with this observation, contemporary utility assessments enroll both patients and health care providers [20-21]. The target sample size of the study was 50 total subjects, which was calculated to allow a 95% confidence interval of ± 0.11 around a mean utility estimate with 89% power [22].    

The survey was developed by the authors based on previously published utility assessments using the Standard Gamble (SG) technique [20-21]. It began with questions on basic demographic information (age, sex, race, marital status, education, and income) and then moved on to SG questions, which are designed to obtain preference weights for seven different health states [23]. The health state descriptions used in the SG were pilot-tested on both health care professionals (n = 2) and patients (n = 3) and modified based on feedback for clarity and ease of use prior to implementation. Five disease-related health states were assessed as well as two additional health states describing serious toxicity associated with GKRS and WBRT. The health states included GKRS, WBRT, salvage WBRT, progression after WBRT, neurologic dying, radionecrosis, and cognitive decline. Health states descriptions used in our survey are in Table 1. Health states were presented in random order. The SG questions asked subjects to imagine they had the condition described in the health state and were then given a choice between two options: remaining in the hypothetical health state or taking a gamble with a new treatment. The gamble had two possible outcomes: full health (with a probability of occurrence *p*) or immediate death (with probability 1 - *p*). The probability *p* was varied in 5% increments until subjects were unable to make a clear choice between remaining in the described health state and trying the new treatment. The goal was to derive the probability *p* associated with the indifference point (i.e., utility) between the certain, current health state and risky alternative treatment. All subjects were surveyed using an encrypted iPad and were surveyed once. Patients enrolled in the study were surveyed immediately following their initial consultation for GKRS and/or WBRT. 

**Table 1:** Health State Descriptions

Name	Description
Gamma Knife Radiosurgery	You undergo Gamma Knife radiosurgery treatment for the cancer in your brain, which is an outpatient procedure requiring one day of treatment. The day includes fitting a head frame, undergoing an MRI, and waiting during treatment planning. The treatment itself involves lying down and the head frame will be secured to the Gamma Knife bed, which slides into the Gamma Knife machine. The treatment takes one to several hours and is not painful. You are not expected to move while in the Gamma Knife machine. Expected side effects include pain/discomfort from the head frame screws as well as fatigue. Other less common side effects include headache, nausea, numbness, hair loss near treated area, seizures, weakness, loss of balance, and vision problems. Following Gamma Knife radiosurgery, you undergo brain imaging every six weeks to see if your cancer has responded to treatment. There is a chance that cancer will return in the brain, requiring treatment with whole brain radiation therapy.
Whole Brain Radiation Therapy	You undergo whole brain radiation therapy for cancer in your brain. This involves lying on a table in an open treatment room and wearing a fitted mask, which is secured to the table, for about 15 minutes. Radiation is given every day, Monday - Friday, over two weeks. Side effects include fatigue, headaches, nausea/vomiting, skin irritation hair loss, and sore throat. Other less common side effects include numbness, hair loss near treated area, seizures, weakness, loss of balance, and vision problems. There are no further treatments available if the cancer does progress after whole brain radiation therapy.
Salvage Whole Brain Radiation Therapy after Gamma Knife Radiosurgery	A routine follow-up MRI after Gamma Knife, unfortunately, shows new disease in the brain that requires additional treatment. Whole brain radiation therapy is recommended. This involves lying on a table in an open treatment room and wearing a fitted mask, which is secured to the table, for about 15 minutes. Treatment is not painful and you are expected to lie still while on the table. Radiation is given every day, Monday - Friday, over two weeks. Side effects include fatigue, headaches, nausea/vomiting, skin irritation hair loss, and sore throat. Other less common side effects include numbness, hair loss near treated area, seizures, weakness, loss of balance and vision problems. There are no further treatments available if the cancer does progress after whole brain radiation therapy.
Progression after Whole Brain Radiation Therapy	Despite treatment, the cancer in your brain progresses. There are no further treatments available. Care is now focused on your quality-of-life and comfort. This means that care is focused on your symptoms (such as controlling pain or nausea) rather than treating the cancer. At this stage, you are terminally sick and unable to care for yourself or participate in your usual activities.
Neurologic Dying	The cancer in your brain continues to progress. You develop neurologic symptoms, including numbness and weakness of arms/legs, headaches, and seizures requiring hospitalization and/or hospice (end of life) care. You are mostly unconscious and bed-bound.
Cognitive Decline	As a result of whole brain radiation, you become increasingly forgetful. For example, you have difficulty remembering where you left your keys or if you took your medication this morning. You also experience poor appetite, sleepiness, and lack of energy. Over time, you develop problems thinking clearly, difficulty doing things you previously found easy, have worsening memory, confusion, headaches, and personality changes. Ultimately, you have increased need for assistance with your activities of daily living, such as dressing yourself, bathing, and cooking.
Radionecrosis	As a result of Gamma Knife radiosurgery, there is damage and swelling of the brain tissue around the tumor, requiring brain surgery to remove it. This involves being admitted to the hospital and undergoing a surgery where an opening is made in the skull in order to access the brain. This will require being in the hospital for several days after surgery. Complications include a very small (< 1%) risk of death, as well as low risks of infection, clots, seizures, or neurologic symptoms.

Descriptive statistics including means, standard deviations, medians, and interquartile ranges (IQR) were generated for demographic variables and health state utilities. Normality was assessed through visual inspection as well as the Skewness-Kurtosis test and showed that the utility distributions were not normally distributed (data not shown). Bivariate analysis of demographic characteristics between patients and nurses were performed using the Chi-square test for categorical variables and the Kruskal-Wallis nonparametric one-way analysis of variance for continuous variables. Pairwise comparisons using the Wilcoxon signed-rank test were used to compare potential differences in overall median utility scores. The Wilcoxon rank-sum test was used to perform subgroup analyses, grouping the cohort by patient/nurse stats, sex, and age (dichotomized by the median value). All statistical analyses were performed with Stata version 13.1 (StataCorp). Statistical tests were two-tailed with α = .05. 

## Results

There were 55 subjects, including 24 patients and 31 nurses, that enrolled at The Yale Cancer Center from December 2013 to May 2015. All subjects completed the survey. Demographic characteristics are summarized in Table 2. The median age in the overall cohort was 51 (range: 23 – 82), and therefore, age was dichotomized as ≤ 50 and > 50 for subgroup analyses. Nurses were more likely to be less than 50 (p = 0.014), female (p = 0.001), and college educated (p < 0.001).

**Table 2:** Subject Characteristics

	Patients (n = 24)			Nurses (n = 31)		P value
Characteristic	No	%		No	%	
Age, Median, IQ range	58 (49.5 – 66.5)			48 (33 – 57)		0.002
Age, Categories						0.014
≤ 50	6		25	18	58	
> 50	18		75	13	42	
Sex						0.001
Male	13		54	4	13	
Female	11		46	27	87	
Race						0.32
White	17		71	25	81	
Black	4		17	1	3	
Latino	1		4	3	10	
Other	2		8	2	6	
Marital Status						0.37
Married	11		46	18	58	
Not Married	13		54	13	42	
Education						< 0.001
College	10		42	31	100	
Non-College	14		58	0	0	
Income						0.12
≤ $49,999	3		13	0	0	
$50,000 - $99,999	13		54	18	58	
≥ $100,000	8		33	13	42	

Utility values are summarized in Table 3. The lowest (i.e., least preferred) median utility elicited was for neurologic dying (0.25, IQR 0.15 – 0.30), indicating that patients would, on average, risk a 25% chance of being dead to avoid experiencing a neurologic death. The next least preferred states were cognitive decline (0.30, IQR 0.20 – 0.40), progression after WBRT (0.40, IQR 0.30 – 0.50), radionecrosis (0.50, IQR 0.40 – 0.60), and salvage WBRT (0.55, IQR 0.45 – 0.65). The highest median utilities, i.e., most preferred states, were for GKRS (0.85, IQR 0.70 – 0.90) and WBRT (0.70, IQR 0.50 – 0.80). The largest variance was seen in radionecrosis among the nurses (median: 0.50) with an IQR of 0.25 and a standard deviation of 21.

**Table 3:** Utility Values for Patients (n = 24), Nurses (n = 31), and Combined (n = 55) GKRS: Gamma Knife radiosurgery, WBRT: whole brain radiation therapy

		Median	Interquartile range	Mean	Standard deviation
GKRS	Patients	0.85	0.75 – 0.90	82	12
	Nurses	0.85	0.70 – 0.88	78	11
	Combined	0.85	0.70 – 0.90	80	12
WBRT	Patients	0.70	0.50 – 0.80	68	14
	Nurses	0.65	0.60 – 0.75	65	16
	Combined	0.70	0.50 – 0.80	66	15
Salvage WBRT	Patients	0.53	0.45 – 0.68	57	14
	Nurses	0.55	0.40 – 0.65	53	16
	Combined	0.55	0.45 – 0.65	54	15
Progression after WBRT	Patients	0.40	0.30 – 0.53	44	14
	Nurses	0.40	0.30 – 0.50	40	15
	Combined	0.40	0.30 – 0.50	42	15
Neurologic Dying	Patients	0.25	0.20 – 0.30	26	9
	Nurses	0.20	0.10 – 0.30	21	11
	Combined	0.25	0.15 – 0.30	23	10
Radionecrosis	Patients	0.50	0.40 – 0.60	52	14
	Nurses	0.50	0.40 – 0.65	51	21
	Combined	0.50	0.40 – 0.60	51	18
Cognitive Decline	Patients	0.35	0.30 – 0.40	35	12
	Nurses	0.30	0.20 – 0.40	32	16
	Combined	0.30	0.20 – 0.40	33	14

Pairwise comparisons revealed that the combined median utility values for each health state were all significantly different from one another. For example, the median value for GKRS was significantly higher (0.85) than the median value of WBRT (0.70, p < 0.001; Table 4). Subgroup analyses were then performed and demonstrated revealed no statistically significant differences between utility values among patients vs. nurses, males vs. females, and for ≤ 50 and > 50 (Table 5).

**Table 4:** Pairwise Comparison of Median Utility Values, Wilcoxon Signed-Rank Test GKRS: Gamma Knife radiosurgery, WBRT: whole brain radiation therapy

	WBRT	Salvage WBRT	Progression after WBRT	Neurologic Dying	Radionecrosis	Cognitive Decline
GKRS	P < 0.001	P < 0.001	P < 0.001	P < 0.001	P < 0.001	P < 0.001
WBRT		P < 0.001	P < 0.001	P < 0.001	P < 0.001	P < 0.001
Salvage WBRT			P < 0.001	P < 0.001	P < 0.006	P < 0.001
Progression after WBRT				P < 0.001	P < 0.001	P < 0.001
Neurologic Dying					P < 0.001	P < 0.001

**Table 5:** Subgroup Analyses, Wilcoxon Rank-Sum Test GKRS: Gamma Knife radiosurgery, WBRT: whole brain radiation therapy

	Patient vs Nurse	Male vs Female	Age > 50 vs ≤ 50
GKRS	P = 0.14	P = 0.65	P = 0.49
WBRT	P = 0.58	P = 0.53	P = 0.87
Salvage WBRT	P = 0.47	P = 0.99	P = 0.64
Progression after WBRT	P = 0.41	P = 0.67	P = 0.61
Neurologic Dying	P = 0.16	P = 0.33	P = 0.12
Radionecrosis	P = 0.94	P = 0.59	P = 0.73
Cognitive Decline	P = 0.49	P = 0.78	P = 0.69

## Discussion

This study provides valuable information about patient preferences for commonly occurring health states for patients with brain metastases. We found that the utility for WBRT (0.70) was significantly lower than GKRS (0.85, P < 0.001). Patients and nurses with direct knowledge of brain metastases perceived neurocognitive complications (such as cognitive decline, radionecrosis, and dying of neurologic progression) as being associated with very low health utilities. This is the first study to directly measure utilities for these health states and can inform future comparative analyses.

Utilities are an integral component of a cost-effectiveness analysis, which plays an increasingly important role in the assessment of emerging technologies. The American Society of Clinical Oncology recently published a guidance statement on the rising cost of cancer care and argues that physicians have a societal responsibility to avoid expensive medical tests and treatments that are not evidence-based in order to minimize health care expenditures [24]. The current study is critical for future comparative and cost-effectiveness studies comparing forms of SRS—including GKRS and CyberKnife—and WBRT. For example, there are several emerging studies examining neurocognitive outcomes with hippocampal-sparing WBRT; however, these rely on provider estimates of utility scores rather than directly quantified utilities using a validated technique, as presented in the current study [25-29]. 

A notable finding in this study is the relatively low median utility score for the cognitive decline health state (0.30). This utility, which was strikingly low, fell between progression after WBRT (0.40) and neurologic dying (0.25). This result emphasizes the importance of perceived functional status and quality of life at the end of life among both patients and nurses who participated in this study. The magnitude of disutility associated with cognitive decline is important to consider when discussing treatment options for patients with brain metastases. Our finding is consistent with previous studies, which demonstrated that the benefits of SRS are largely driven by improvements in neurocognitive outcomes rather than survival [3-5, 19]. The differential impact of SRS and WBRT on cognitive function should be a focal point of discussion during treatment decisions due to its potential impact on patients’ quality of life. 

The literature is mixed as to whether utility values elicited from patients differ from non-patients, such as health care providers. Generally, patient utility values are often higher, which is thought to be due to patient adaptation to morbidity [30]. In our study, patients reported slightly higher median values than nurses (e.g., 0.70 vs. 0.65 for WBRT); however, these estimates were not statistically different from one another. Therefore, we recommend using the combined median scores for utility values.

The findings of this study may not be generalizable to people outside of our cohorts, that is, patients with brain metastases and oncology nurses with the demographics summarized in Table 2. Nonetheless, we observed no statistically significant difference in utility values between patients and nurses. Therefore, we feel these utility values should be applicable to the majority of patients with brain metastases. Although we used patient and provider feedback from the pilot test to inform the health state descriptions, the descriptions (Table 1) still may not represent the average experience for patients undergoing radiation therapy for brain metastases. Finally, while our study was sufficiently powered to detect differences in utility values in the overall cohort, our sample size may have been too small to detect meaningful differences across demographic variables (subgroups).

## Conclusions

Our study quantifies health state utility values for brain metastases-related health states. The findings inform clinical decision making by quantifying and providing directly measured perceptions of the impact of therapy and disease on quality-of-life. Our results support the shift towards more focal treatment for brain metastases; however, the potential benefit is likely also dependent on the patient's prognosis. Furthermore, these utility values can be used in decision analyses and cost-effectiveness studies that are critically needed to evaluate emerging techniques with WBRT and/or SRS. 
